# Design and Calibration of a Single-Lens Telecentric Four-Camera Array Based on Planar Mirrors and Its Application in Strain Measurement

**DOI:** 10.3390/s26051427

**Published:** 2026-02-25

**Authors:** Xu Zhang, Guo Chen

**Affiliations:** 1 College of Civil Aviation, Nanjing University of Aeronautics and Astronautics, Nanjing 210016, China; nuaa_ides_zx@nuaa.edu.cn; 2 College of General Aviation and Flight, Nanjing University of Aeronautics and Astronautics, Liyang 213300, China

**Keywords:** telecentric imaging, virtual camera array, perspective error elimination, precision metrology, pipeline assembly

## Abstract

As the primary power transmission conduits, aircraft hydraulic pipelines are critical for actuating flight control surfaces and landing gear systems. Accurate in situ strain evaluation of these pipelines is essential, as installation-induced pre-loads directly compromise fatigue life and sealing performance, threatening overall system reliability. However, such evaluation is frequently hindered by the perspective distortions and limited depth of field inherent in conventional imaging systems. To overcome these metrological limitations, this study presents a novel virtual telecentric camera array system designed for high-precision, non-contact strain measurement. Unlike traditional pinhole models, the proposed system leverages a catadioptric setup with planar mirrors to create a virtual four-eye telecentric array from a single physical lens, ensuring constant magnification within the depth of field. A comprehensive simulation framework was established to rigorously compare the reprojection errors and scale accuracies between telecentric and pinhole projection models, quantitatively demonstrating the superior stability of the telecentric approach. Furthermore, a dedicated calibration strategy for non-overlapping telecentric fields of view was developed and validated. Experimental results from pipeline installation tests indicate a high concordance with strain gauge data, confirming that the proposed telecentric system effectively mitigates parallax errors and provides a robust solution for static and quasi-static micro-scale deformation monitoring in complex assembly environments.

## 1. Introduction

### 1.1. Background and Motivation

The operational integrity of aircraft hydraulic systems is intrinsically linked to the geometric compliance of their pipeline networks. These pipelines serve as the ‘arteries’ of the aircraft, delivering high-pressure hydraulic power to essential actuators such as flight control surfaces and landing gears. During the rigid coupling of these components, even minor dimensional discrepancies—such as angular misalignments or axial gaps—can introduce substantial pre-loads into the structure. These “installation strains” act as a persistent static load, which, when superimposed with flight-induced vibrations, significantly reduces the fatigue and life of joints and sealing interfaces [1,2,3]. Therefore, ensuring the stress-free assembly of hydraulic lines is not merely a manufacturing requirement but a critical safeguard for flightworthiness.

Existing methodologies for pipeline status assessment have predominantly relied on mechanical vibration analysis and contact-based sensing. Early investigations, such as those by the research team led by Chen Guo, successfully implemented hammer impact modal analysis to detect qualitative anomalies in installation states [4,5]. However, this global response method inherently lacks the spatial resolution to quantify localized strain distributions. Conversely, traditional localized sensors like resistance strain gauges and extensometers are regarded as the gold standard for precision [6]. Yet, their deployment in an aircraft assembly environment is plagued by severe logistical constraints. The mandatory surface abrasion and adhesive curing processes are not only labor-intensive but also irreversible, potentially compromising the anti-corrosive coatings of the pipeline. Furthermore, the physical cabling required for these sensors interferes with the complex assembly workflow, rendering them unsuitable for rapid, in situ compliance checks. While Fiber Bragg Grating (FBG) sensors offer immunity to electromagnetic interference, the inherent fragility of optical fibers poses significant integration challenges [7,8]. Bonding these rigid, brittle fibers onto the small-radius curvature of hydraulic tubes often leads to signal attenuation or structural detachment, limiting their robust application in compact aerospace layouts.

Non-destructive testing (NDT) technologies based on electromagnetic and acoustic principles have also been scrutinized for this application. Eddy current testing, which correlates stress with conductivity changes, theoretically offers a non-contact solution [9]. However, its efficacy is fundamentally constrained by the material properties of modern aerospace pipelines. Since these components are typically fabricated from non-ferromagnetic alloys (e.g., titanium or stainless steel), the magnetic permeability response is negligible. Moreover, the “lift-off effect”—where signal sensitivity fluctuates drastically with the distance between the probe and the curved surface—introduces unacceptable measurement uncertainty on small-diameter tubes [10]. Similarly, ultrasonic stress measurement relies on the acoustoelastic effect, where wave velocity shifts with stress [11,12]. Although promising for large structural components, this technique remains experimental for thin-walled hydraulic pipes. The coupling instability on high-curvature surfaces and the complex wave dispersion in thin walls make signal interpretation notoriously difficult, preventing its transition from laboratory research to engineering practice.

In response to these limitations, vision-based metrology, particularly Digital Image Correlation (DIC), has emerged as a potent alternative due to its full-field capability [13,14]. However, applying standard computer vision techniques to small-scale aircraft pipelines introduces a specific optical dilemma. Conventional industrial cameras utilize entocentric (pinhole) lenses, which are subject to the laws of perspective projection: the magnification of an object changes inversely with its distance from the lens. In a dynamic assembly site, even minute vibrations or positioning errors along the optical axis (*Z*-axis) will cause the pipeline to appear larger or smaller, introducing artificial “strains” into the measurement data [15]. For small-sized components requiring micro-strain accuracy, these perspective-induced errors are fatal. To overcome this fundamental optical barrier, this study proposes a paradigm shift from pinhole to telecentric imaging. Unlike standard lenses, object-space telecentric lenses filter out the principal rays that are not parallel to the optical axis, ensuring strictly orthographic projection and maintaining constant magnification regardless of object displacement within the depth of field [15].

### 1.2. Problem Statement and Application Scenario

**Figure 1:**
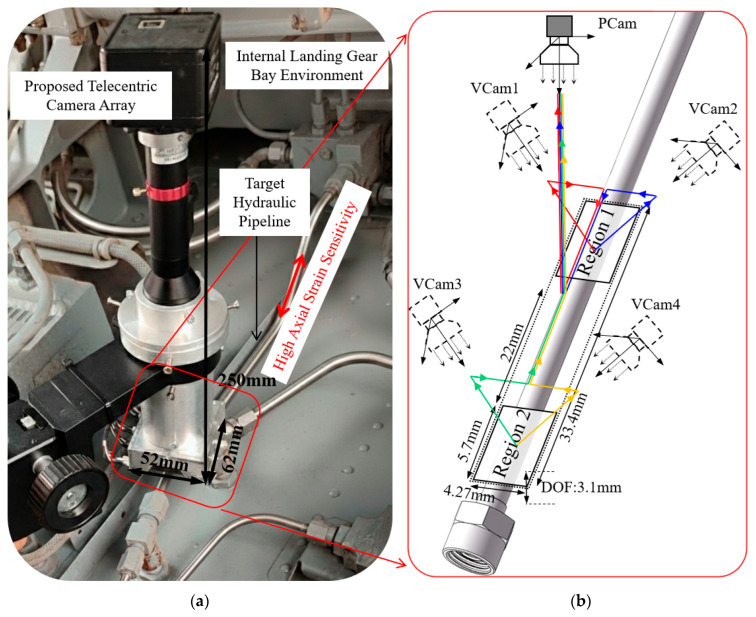
System deployment and optical principle: (**a**) Real-world installation scenario within an internal landing gear bay, highlighting the compact design for constrained spaces; (**b**) Schematic of the virtual telecentric camera array, illustrating the transformation from a single physical camera (PCam) to four virtual viewpoints (VCam1–4) for enhanced 3D axial strain sensitivity. The arrows of different colors indicate the four distinct optical paths corresponding to these virtual viewpoints.

The proposed virtual telecentric system is specifically engineered for in situ measurement during the final assembly and rigid coupling stages of aircraft hydraulic systems. As illustrated in the real-world deployment scenario in Figure 1a, the measurement is intended to be performed within the highly constrained internal compartments of the airframe, such as the internal landing gear bay environment. In these spaces, the system must accurately quantify installation-induced pre-loads while being mounted in close proximity to the pipeline joints, where traditional large-scale vision systems are impractical.

To overcome the challenges of manual installation and mounting position uncertainty, the system leverages a virtual telecentric camera array (Figure 1b). By transforming a single physical camera (PCam) into four virtual viewpoints (VCam1–4), the system achieves precise 3D spatial position recognition through binocular stereo vision. This optical configuration is specifically optimized for high axial strain sensitivity within a 33.4 mm field of view, ensuring reliable monitoring of pipeline integrity even when subjected to *z*-axis positioning fluctuations within the 3.1 mm depth-of-field (DOF) range.

### 1.3. Proposed Method and Paper Contributions

**Figure 2:**
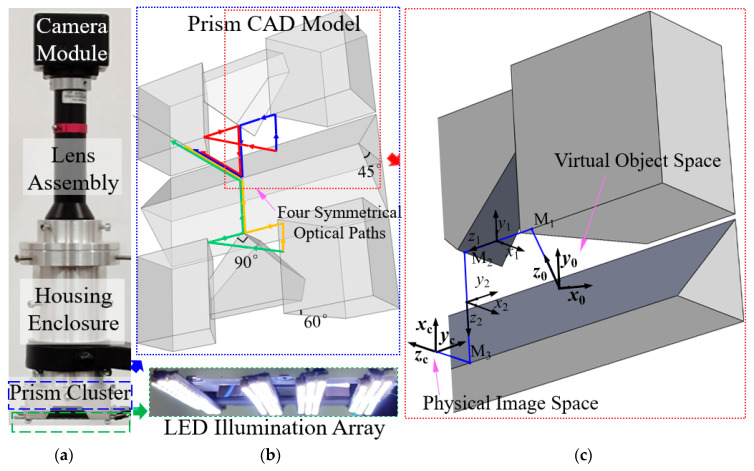
Structural composition and optical path modeling: (**a**) Physical assembly of the telecentric camera system; (**b**) CAD model of the prism cluster illustrating the four-way optical axis splitting; (**c**) 3D ray-tracing of a single sub-view path, showing the coordinate system evolution (*x_i_*, *y_i_*, *z_i_*) through three successive reflections from virtual object space (0) to physical image space (**c**). The arrows of different colors represent the four distinct optical paths generated by the prism cluster.

To address the critical issue of inaccurate strain measurement following aircraft hydraulic pipeline installation, this study proposes a virtual telecentric camera array system based on a customized catadioptric design. As shown in the structural composition in Figure 2a, the system integrates an industrial camera module, a telecentric lens assembly, and a customized mirror housing enclosure with a prism cluster at its core. To ensure stable imaging in dark compartments, a high-intensity LED illumination array is incorporated at the base.

The optical path is engineered through a hierarchical beam-splitting structure (Figure 2b). As detailed in the 3D ray-tracing model in Figure 2c, the system directs the optical axis through three successive reflections to form the virtual array. The coordinate system evolves from the virtual object space (*x*_0_, *y*_0_, *z*_0_) through intermediate reflection states (*x*_1_, *y*_1_, *z*_1_) and (*x*_2_, *y*_2_, *z*_2_), finally reaching the physical image space (*x_c_*, *y*_c_, *z_c_*). This external surface reflection approach effectively eliminates chromatic aberration associated with glass transmission, while the prism geometry ensures rigorous structural stability without complex adjustment mechanisms.

While telecentric imaging resolves the perspective error, creating a multi-view array introduces complex calibration requirements. Specifically, measuring long pipeline sections necessitates splitting the field of view into non-overlapping regions. Calibrating such disjointed systems is a recognized challenge in photogrammetry [16]. Although mirror-based virtual camera calibration [17,18] and refractive prism models [19] have been explored for pinhole systems, methodologies tailored for catadioptric telecentric arrays remain sparse. Standard planar targets often fail due to the lack of common features between views and the depth-insensitive nature of telecentric projection. To address this, this study develops a comprehensive calibration framework using a large-scale target specifically matched to the telecentric array, ensuring rigorous global optimization of the extrinsic parameters for high-precision in situ strain assessment.

The main research work of this paper includes: (1) Constructing a virtual telecentric camera array system, completing the image acquisition and calibration process through simulation, and conducting a comparative analysis with the traditional pinhole model. The results show that the telecentric model has smaller reprojection error and better scale measurement accuracy; (2) Building a physical telecentric camera array system, conducting experimental calibration and scale measurement error analysis, and verifying that the actual measurement accuracy of the system meets the expected design goals; (3) Designing and carrying out pipeline installation strain test experiments, comparing the measurement results of the telecentric camera array with the strain gauge measurement data. The high consistency between the two proves the feasibility and reliability of this method in the static and quasi-static in situ strain monitoring of aircraft hydraulic pipelines.

## 2. Calibration Methodology and Mathematical Formulation

### 2.1. Intrinsic Calibration of Telecentric Camera

#### 2.1.1. Telecentric Projection Model

Telecentric projection is essentially a type of orthographic projection, and its projection relationship is shown in Equation (1). In the equation, *K′* is a 3 × 4 intrinsic parameter matrix, and all elements in the 3rd column of this matrix are 0; generally, the *z*-axis component of the feature point *P_w_′* on the calibration target is 0. After simplifying Equation (1), the intrinsic parameter matrix K and the partial components of the rotation-translation matrix [*R_s_*|*t_s_*] can be obtained. The multiplication of these two matrices yields the homography matrix H with 6 parameters.

Distortion occurs in the xy-plane of the camera coordinate system: Let *X_c_*, *Y_c_* be the coordinates of the feature point (transformed from the world coordinate system) in the camera coordinate system. After correcting this coordinate via Equation (2), the undistorted coordinate is obtained. Multiplying this undistorted coordinate by the intrinsic parameter matrix *K* gives the final pixel coordinate. Practice has shown that a 5-parameter distortion model can effectively correct most common distortion scenarios; to simplify the subsequent formula derivation process, the undistorted coordinate is used in the following text.

(1) $$\begin{array}{l} \;\;\;p_{uv}=K^{\prime }\left[R|t\right]P_{w}^{\prime }\\ \left(\begin{array}{l} u\\ v\\ 1 \end{array}\right)=\left(\begin{array}{c} m_{x}\\ 0\\ 0 \end{array}\begin{array}{c} 0\\ m_{y}\\ 0 \end{array}\begin{array}{c} 0\\ 0\\ 0 \end{array}\begin{array}{c} c_{x}\\ c_{y}\\ 1 \end{array}\right)\left(\begin{array}{cccc} r_{11} & r_{12} & r_{13} & t_{1}\\ r_{21} & r_{22} & r_{23} & t_{2}\\ r_{31} & r_{32} & r_{33} & t_{3}\\ 0 & 0 & 0 & 1 \end{array}\right)\left(\begin{array}{c} X_{w}\\ Y_{w}\\ Z_{w}=0\\ 1 \end{array}\right)\\ \;\;\;\;\;\;\;\;\;\;\;\;=\left(\begin{array}{c} m_{x}\\ 0\\ 0 \end{array}\begin{array}{c} 0\\ m_{y}\\ 0 \end{array}\begin{array}{c} c_{x}\\ c_{y}\\ 1 \end{array}\right)\left(\begin{array}{ccc} r_{11} & r_{12} & t_{1}\\ r_{21} & r_{22} & t_{2}\\ 0 & 0 & 1 \end{array}\right)\left(\begin{array}{c} X_{w}\\ Y_{w}\\ 1 \end{array}\right)\\ \;\;\;\;\;\;\;\;\;\;\;\;=K\left[R_{s}|t_{s}\right]P_{w}\\ \;\;\;\;\;\;\;\;\;\;\;\;=\left(\begin{array}{ccc} h_{11} & h_{12} & h_{13}\\ h_{21} & h_{22} & h_{23}\\ 0 & 0 & 1 \end{array}\right)P_{w}\\ \;\;\;\;\;\;\;\;\;\;\;\;=HP_{w} \end{array}$$

(2) $$\begin{array}{l} \left\{\begin{array}{c} X_{c,distort}=X_{c}(1+k_{1}r^{2}+k_{2}r^{4}+k_{3}r^{6})+2p_{1}X_{c}Y_{c}+p_{2}(r^{2}+2X_{c}^{2})\\ Y_{c,distort}=Y_{c}(1+k_{1}r^{2}+k_{2}r^{4}+k_{3}r^{6})+p_{1}(r^{2}+2Y_{c}^{2})+2p_{2}X_{c}Y_{c}\;\;\;\; \end{array}\right.\\ r^{2}=X_{c}^{2}+Y_{c}^{2} \end{array}$$

#### 2.1.2. Initialization of Parameters

In a single calibration target image captured by a telecentric camera, the feature points of the calibration target lie on the same physical plane, and their corresponding pixel points are also distributed on the same image plane. The mapping relationship of feature points between the two planes can be described by the homography matrix *H*. Specifically, the *H* matrix is solved by establishing a system of linear equations and using the Direct Linear Transformation (DLT) method; the subsequent process of solving the initial value of the intrinsic parameter matrix *K* based on *H* is as follows:

The expression of $R_{s}^{T}$ ∗ *R_s_* can be derived from Equation (3), and the determinant property of $R_{s}^{T}$ ∗ *R_s_* can be obtained from Equation (4); combining these two equations, the correlation equation between *H* and *K* (shown in Equation (5)) can be established. Multiple homography matrices *H* are obtained by collecting multiple sets of calibration target images, and a system of linear equations is constructed based on the data of these matrices. Finally, the horizontal magnification *m_x_* and vertical magnification *m_y_* of the telecentric camera are solved via Equation (6).

(3) $$\;{\left(A^{-1}H\right)}^{T}A^{-1}H=\left(\begin{array}{cc} R_{s}^{T}R_{s} & R_{s}^{T}T_{s}\\ T_{s}^{T}R_{s} & T_{s}^{T}T_{s}+1 \end{array}\right)$$

(4) $$\begin{array}{l} \;R_{s}^{T}R_{s}-I_{2\times 2}=\left(\begin{array}{cc} 1-r_{31}^{2} & -r_{31}r_{32}\\ -r_{31}r_{32} & 1-r_{32}^{2} \end{array}\right)-I_{2\times 2}\\ \;\;\;\;\;\;\;\;\;\;\;\;\;\;\;\;\;\;\;\;\;\;=\left(\begin{array}{cc} -r_{31}^{2} & -r_{31}r_{32}\\ -r_{31}r_{32} & -r_{32}^{2} \end{array}\right)\\ \det(R_{s}^{T}R_{s}-I_{2\times 2})=0 \end{array}$$

(5) $$\begin{array}{l} \;l_{1}-\;l_{2}(h_{21}^{2}+h_{22}^{2})-\;l_{3}(h_{11}^{2}+h_{12}^{2})=-{\left(h_{11}h_{22}-h_{12}h_{21}\right)}^{2}\\ l_{1}-\;l_{2}\;\;\;\;\;\;\;\;\;\;\;\;\;\;G_{1}\;-\;l_{3}\;\;\;\;\;\;\;\;\;\;\;\;\;\;G_{2}=w\\ \;\;\;\;\;\;\;\;\;\;\;\;\;\;\;\;\;\;\;\;\;\;\;\left(\begin{array}{ccc} 1 & G_{1} & G_{2}\; \end{array}\right)\left(\begin{array}{c} l_{1}\\ l_{2}\\ l_{3} \end{array}\right)=w \end{array}$$

(6) $$\begin{array}{l} \;l_{1}=m_{x}^{2}m_{y}^{2};l_{2}=m_{x}^{2};l_{3}=m_{y}^{2};\\ m_{x}=\sqrt{l_{2}};m_{y}=\sqrt{l_{3}}; \end{array}$$

#### 2.1.3. Nonlinear Optimization of Intrinsic Parameters

The initial value of the intrinsic parameters solved in the previous section has certain errors, and it is necessary to perform nonlinear optimization using all calibration data to improve accuracy. The optimization objective is to minimize the reprojection error, as shown in Equation (7). The optimization variables include the intrinsic parameter matrix *K*, distortion coefficient *D*, and the rotation-translation parameters [*R_s_|t_s_*]*_i_* corresponding to each calibration target image. Among them, the initial values of the principal point coordinates (*c_x_*,*c_y_*) in the intrinsic parameter matrix and the distortion coefficient *D* are both set to 0 to simplify the initial process of optimization iteration.

(7) $$\begin{array}{l} f(x)=\min\frac{1}{2}\sum_{i=0}^{n-1}\sum_{j=0}^{m-1}∥p_{ij}-\tilde{p}(K,{\left[R_{s}|t_{s}\right]}_{i},D,P_{j}){∥}_{2}^{2}\\ x={\left(K,D,{\left[R_{s}|t_{s}\right]}_{i}\right)}_{i=0\sim n-1}\\ s.t.\left\{\begin{array}{l} t_{x}=(h_{13}-c_{x})/m_{x}\\ t_{x}=(h_{23}-c_{y})/m_{y}\\ c_{x}^{\left(0\right)}=0;c_{y}^{\left(0\right)}=0\\ D^{\left(0\right)}=[0,0,0,0,0] \end{array}\right. \end{array}$$

where *p_ij_* is the actual pixel coordinate of the *j*-th feature point in the *i*-th image, $\tilde{p}$ is its reprojected pixel coordinate, and *P_j_* is the world coordinate of the *j*-th feature point.

### 2.2. Estimation of Initial Extrinsics for Binocular Pairs

#### 2.2.1. Estimation of Candidate Extrinsics (R, t)

**Figure 3:**
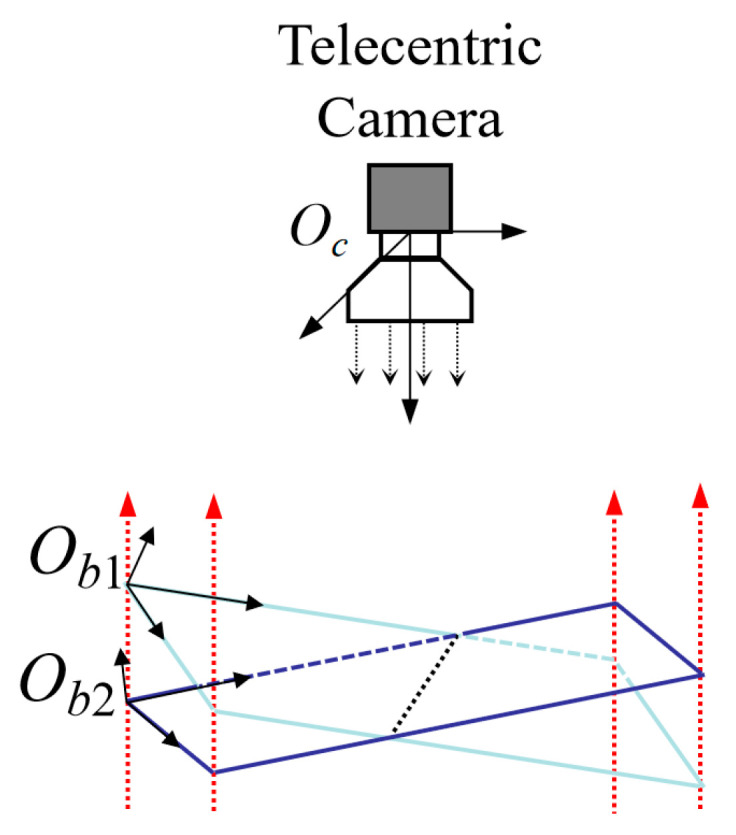
Ambiguity of camera pose estimation in telecentric projection. The red dashed arrows represent the parallel projection rays of the telecentric system, illustrating that points on different planes *O_b_*_1_ and *O_b_*_2_ project to the same image coordinates.

After determining the intrinsic parameter matrix *K* and distortion coefficient *D* of the telecentric camera, the components *r*_13_ and *r*_23_ of the rotation matrix *R_s_* can be calculated via Equation (8). However, there is uncertainty in the sign of the calculation result, meaning there are two possible solutions; Figure 3 shows the phenomenon where two different calibration target positions correspond to the same projection effect under telecentric projection, indicating that there are two possible solutions for the actual camera position, which is consistent with the mathematical property of Equation (8).

(8) $$\left\{\begin{array}{l} r_{13}=\pm \sqrt{1-r_{11}^{2}-r_{12}^{2}}\\ r_{23}=\pm \sqrt{1-r_{21}^{2}-r_{22}^{2}}\\ r_{13}r_{23}=-(r_{11}r_{21}+r_{12}r_{22}) \end{array}\right.$$

#### 2.2.2. Disambiguation Using Multiple Images

Since the feature points on a planar calibration target lack depth information, the actual camera pose cannot be uniquely determined from a single image. To resolve this, a *z*-axis translation stage with a 20 mm stroke is utilized to move the target along the positive *z*-axis. In both simulation and experimental phases, a step size of *Z* + 1 mm is adopted. This specific distance is chosen to ensure that all feature points remain within the common FOV and the effective depth of field (DOF) of the telecentric lens while producing a significant pixel-level displacement for reliable disambiguation. By comparing the projection differences via Equation (9), the signs of *r*_13_ and *r*_23_ are determined. It is important to note that the initial analytical solution for [R|t] typically yields a high reprojection error. To improve stability, a non-linear optimization using the Ceres Solver with the Levenberg–Marquardt (LM) algorithm is implemented. While optimization alone cannot resolve the sign ambiguity, it significantly refines the pose parameters. In cases where the lens is nearly perpendicular to the target, the components *r*_13_ and *r*_23_ approach zero; although this may increase the sensitivity of sign determination under minor vibrations, the impact on the reprojection error remains negligible. Furthermore, this process serves only as an initial pose estimation for individual cameras. The final stability and metric accuracy of the system are strictly guaranteed by the global bundle adjustment (Section 2.4), which optimizes the entire camera array’s extrinsics and measurement consistency.

(9) $$Z_{+}\left(\begin{array}{c} r_{13}\\ r_{23}\\ 1 \end{array}\right)=K^{-1}(\left(\begin{array}{c} u_{+}\\ v_{+}\\ 1 \end{array}\right)-\left(\begin{array}{c} u\\ v\\ 1 \end{array}\right))$$

### 2.3. Global Calibration Strategy for Non-Overlapping FOVs

**Figure 4:**
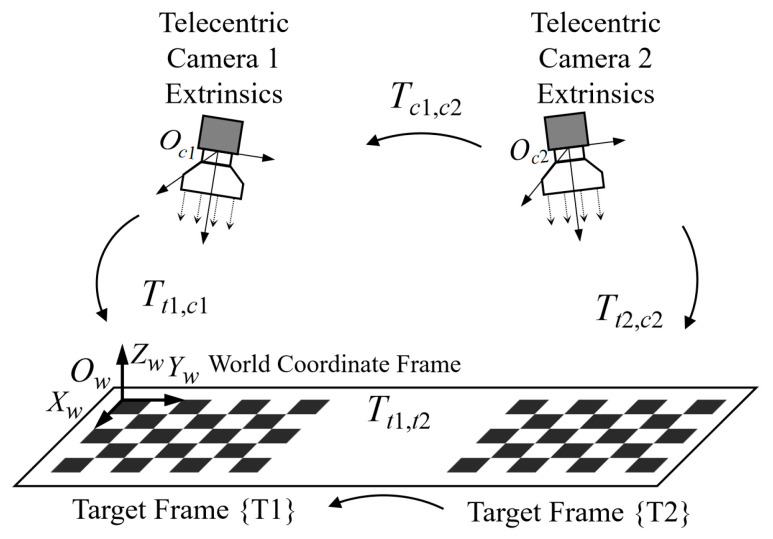
Calibration Target for Non-Overlapping Fields of View.

The calculation process of the extrinsics for the four-camera array is relatively complex, and it is difficult to derive the relevant formulas and express the schematic diagrams. This section focuses only on the method for calculating the extrinsics between two cameras with non-overlapping fields of view. A dedicated calibration target adapted to non-overlapping fields of view (as shown in Figure 4) is used in the experiment, which consists of two sub-calibration targets with known spatial positional relationships.

First, the pose parameters of the two cameras relative to the two sub-calibration targets are calculated separately using the method described in Section 2.2.1; then, using the known spatial transformation relationship between the two sub-calibration targets, the relative pose (extrinsics) between the two cameras can be solved via Equation (10). To completely eliminate the ambiguity of the telecentric camera pose, at least two sets of calibration target images need to be collected in the experiment—in the two sets of images, there is a certain displacement difference of the calibration target along the *z*-axis. Based on this displacement difference, the relative positional relationship between the two cameras can be accurately calculated, avoiding the incomplete disambiguation caused by a single image.

(10) $$\begin{array}{c} T_{t1,c2}=T_{t1,t2}T_{t2,c2}\\ T_{c1,c2}={T_{t1,c1}}^{-1}T_{t1,c2}={T_{t1,c1}}^{-1}T_{t1,t2}T_{t2,c2} \end{array}$$

where *T_c_*_1,*c*2_ is the pose transformation matrix of Camera 2 relative to Camera 1, *T_t_*_2,*c*2_ is the pose matrix of Camera 2 relative to Calibration Target 2, and *T_t_*_1,*c*1_ is the pose matrix of Calibration Target 2 relative to Camera 1.

### 2.4. Global Optimization Based on Bundle Adjustment

#### 2.4.1. Determination of Consistent Extrinsics in the Array

**Figure 5:**
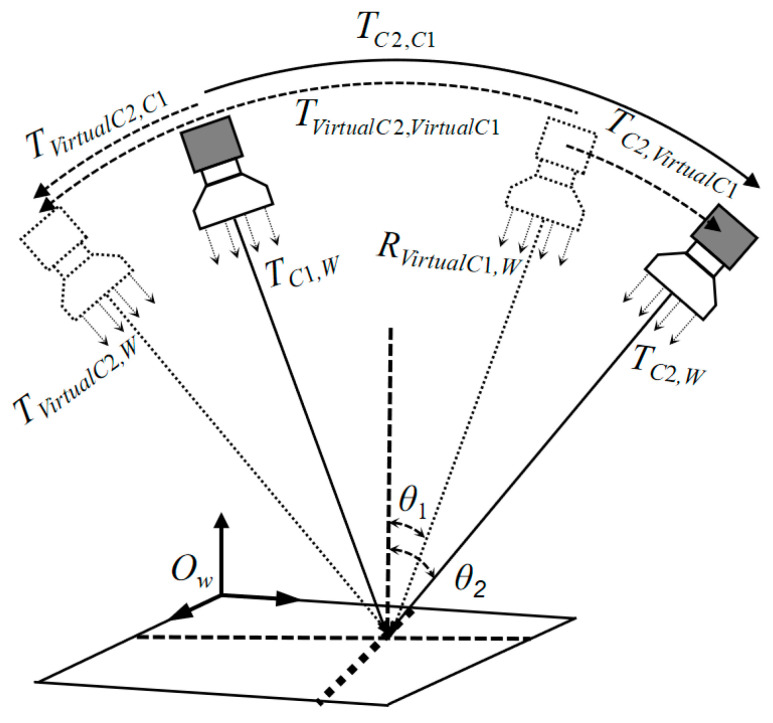
Possible Real and Virtual Camera Positions Relative to the Calibration Board.

After calculating the initial extrinsics between cameras, it is necessary to capture multiple calibration target images and combine them with nonlinear optimization to further improve the parameter accuracy. The calculation of initial extrinsics relies on multiple sets of image pairs. To simplify the operation process, this section first discusses the method of determining the position of the camera coordinate system based on a single image: As shown in Figure 5, this figure shows the possible position distribution of the binocular telecentric camera relative to the calibration target, where the solid lines represent the actual camera positions and the dashed lines represent the virtual camera positions.

By combining the actual cameras and virtual cameras in pairs, 4 sets of inter-camera pose relationships can be obtained; by comparing these combined relationships with the initial inter-camera pose calculated in Section 2.3, the correct pose that conforms to the actual scenario can be screened out, thereby determining the actual position of the camera coordinate system. It should be noted that the actual camera and virtual camera are symmetrically distributed with the calibration target plane as the axis of symmetry, and the axes of symmetry of the binocular cameras (left and right) may differ. For the convenience of display, they are drawn on the same plane in Figure 5.

#### 2.4.2. Bundle Adjustment for Extrinsic Refinement

The Bundle Adjustment method is used for the refined optimization of camera extrinsics, and the optimization objective function is shown in Equation (11). In the equation, *i* represents any one of the n cameras, *j* represents any one of the m calibration target feature points, and *k* represents any one of the *l* measurements; the optimization objective is to minimize the reprojection error of all feature points, and the optimization variables include the relative extrinsics between cameras and the absolute position of the camera coordinate system in each measurement. Through nonlinear iterative solution, more accurate inter-camera extrinsics can be obtained.

(11) $$\begin{array}{l} f(x)=\min\frac{1}{2}\sum_{i=0}^{n-1}\sum_{j=0}^{m}\sum_{k=0}^{l}∥p_{ijk}-\tilde{p}(K_{i},{\left[R_{s}|t_{s}\right]}_{i},D_{i},P_{j},T_{k}){∥}_{2}^{2}\\ x={\left(T_{k},{\left[R_{s}|t_{s}\right]}_{i}\right)}_{k=0\sim l-1,i=0\sim n-1} \end{array}$$

where *p_ijk_* is the actual pixel coordinate of the *j*-th feature point captured by the *i*-th camera in the *k*-th measurement, $\tilde{p}$ is its reprojected coordinate, and [*R_s_*|*t_s_*]*_i_* is the pose matrix of the *i*-th camera relative to the global coordinate system.

The proposed calibration algorithm was implemented in C++ on an Ubuntu 18.04 platform. Specifically, the non-linear least squares problem described in Equation (11) was solved using the Ceres Solver library, employing the Levenberg–Marquardt (LM) algorithm to ensure robust convergence.

## 3. Numerical Validation and Comparative Analysis

To verify the correctness and superiority of the telecentric model, this section uses the telecentric model and the pinhole model to calibrate the simulated captured data respectively. Through the measurement results of the ruler length, the calibration reprojection error (representing internal fitting quality), and the ruler measurement error (representing external measurement accuracy) of the two models are compared.

**Figure 6:**
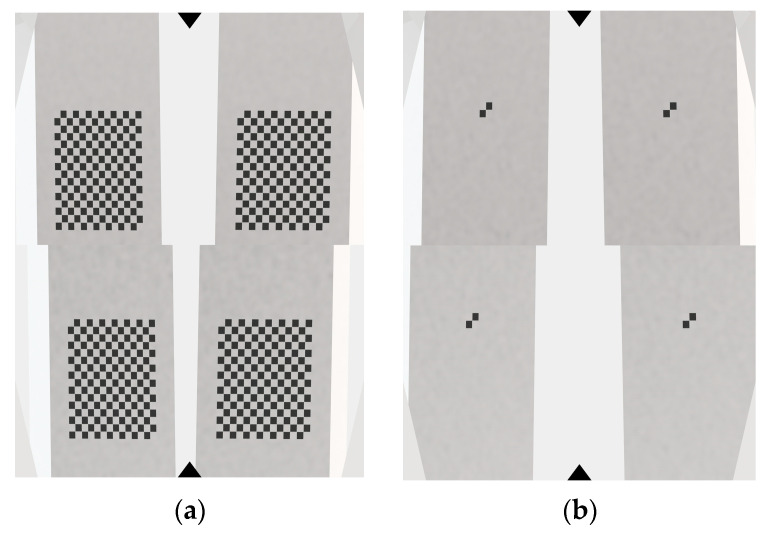
Simulated images. (**a**) calibration board; (**b**) ruler.

**Figure 7:**
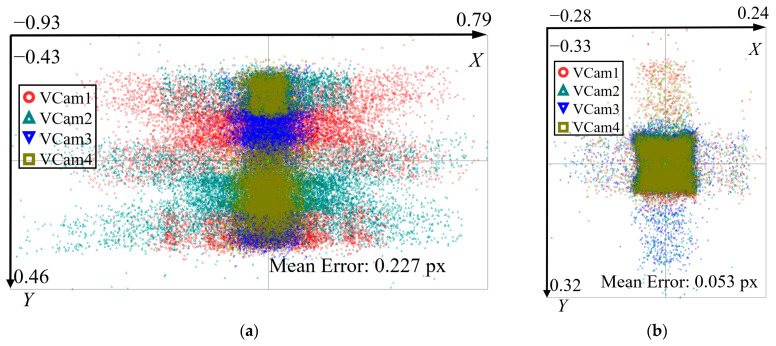
Reprojection error distribution. (**a**) pinhole model; (**b**) telecentric model.

**Figure 8:**
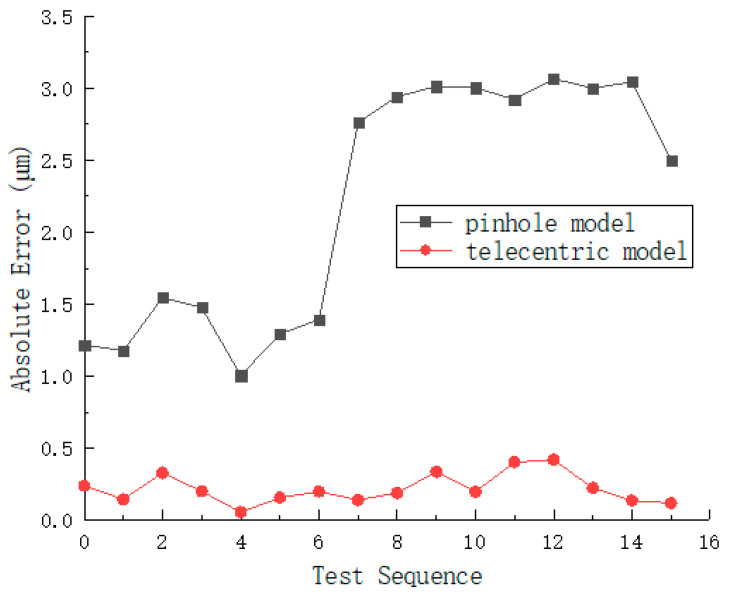
RMSE of ruler length measurement for pinhole and telecentric models.

SolidWorks 2022 software is used to construct a virtual experimental scenario. A virtual calibration target and ruler were modeled with identical geometric parameters to the physical reference standards described in Section 4.1. Specifically, one part of the calibration board consists of a 14 × 10 black-and-white checkerboard containing 13 × 9 feature points, while one part of the ruler consists of a 2 × 2 black-and-white checkerboard containing a single feature point. The binocular angle of the 4 virtual cameras is set to 60°, and the distance between the two camera groups (with each group consisting of binocular cameras) is 22 mm. Based on the secondary development function of SolidWorks, 72 calibration target images are generated (the positions of the calibration targets are evenly distributed and a certain amount of random noise is added to simulate actual capture errors), and 16 ruler images are generated using the same method; as shown in Figure 6, (a) is the calibration target image captured by the four-camera simulation, and (b) is the ruler image captured by the simulation.To evaluate the calibration accuracy, the reprojection error distributions of the proposed telecentric model and the traditional pinhole model were calculated and compared, as shown in Figure 7. Furthermore, the length measurement performance was assessed by analyzing the Root Mean Square Error (RMSE) of the ruler length for both models, as illustrated in Figure 8.

## 4. Experimental Validation and Application in Pipeline Strain Measurement

**Figure 9:**
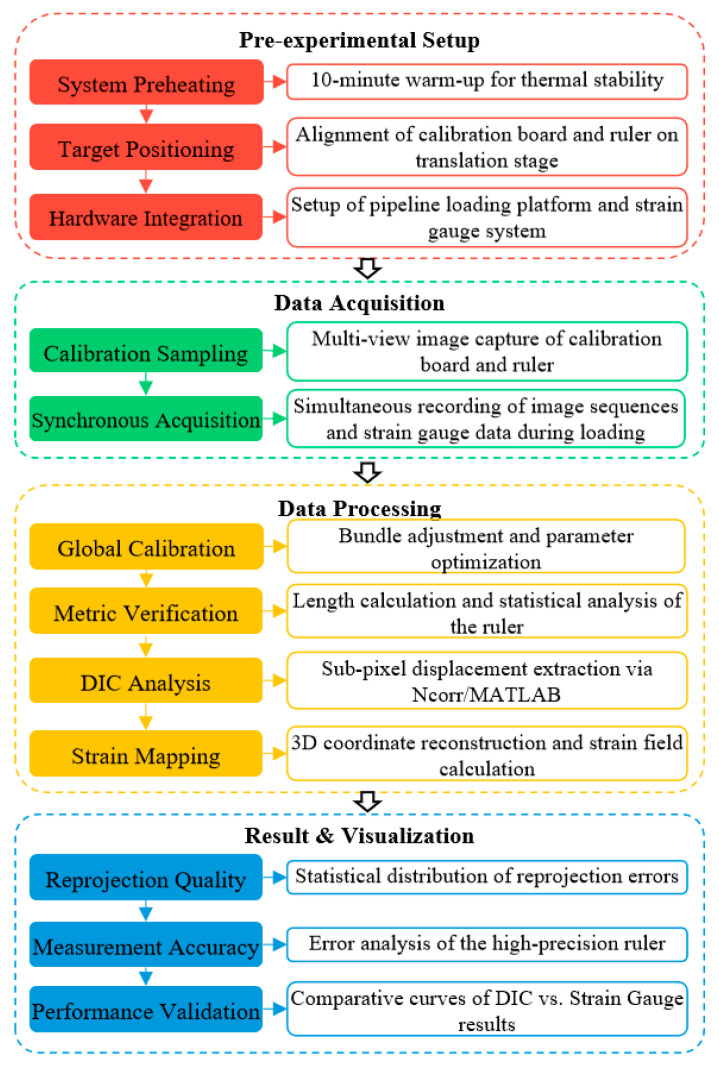
Implementation workflow of the experimental system, illustrating the transition from hardware initialization to final performance validation.

To ensure the metrological traceability and engineering practicalities of the proposed system, the experimental evaluation is conducted in three progressive stages. First, the physical camera array is calibrated to establish its intrinsic and extrinsic parameters. Second, a metric verification is performed using a high-precision independent standard to evaluate the spatial measurement accuracy. Finally, the system is applied to a simulated aircraft pipeline installation to validate its performance against traditional contact-based sensors. The comprehensive implementation workflow, covering data acquisition, processing, and result visualization, is illustrated in Figure 9.

### 4.1. Physical System Calibration and Internal Fitting Quality

#### 4.1.1. Experimental Hardware and Reference Targets

The imaging hardware comprises a YW500 industrial camera (Yangwang, Shenzhen, China) with a resolution of 2592 × 1944 pixels and a pixel size of 2.2 μm, coupled with a 0.5 × bi-telecentric lens. This configuration yields an effective object-side resolution of 4.4 μm/pixel.

**Figure 10:**
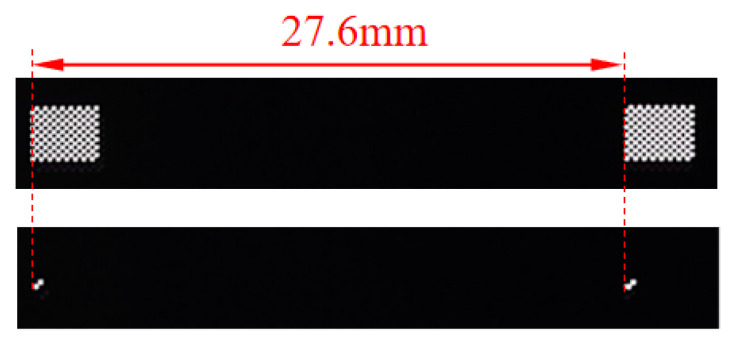
Physical diagram and dimensions of the calibration target and ruler.

To guarantee the reliability of the calibration and measurement references, a high-precision physical calibration target and a ruler were fabricated using the VPG 200 Mask Writer (Heidelberg Instruments, Heidelberg, Germany). As shown in Figure 10, the ruler consists of two high-precision feature points with a center distance of 27.6 mm, and the manufacturing accuracy of both targets reaches ±0.25 μm.

#### 4.1.2. Calibration Process and Error Analysis

**Figure 11:**
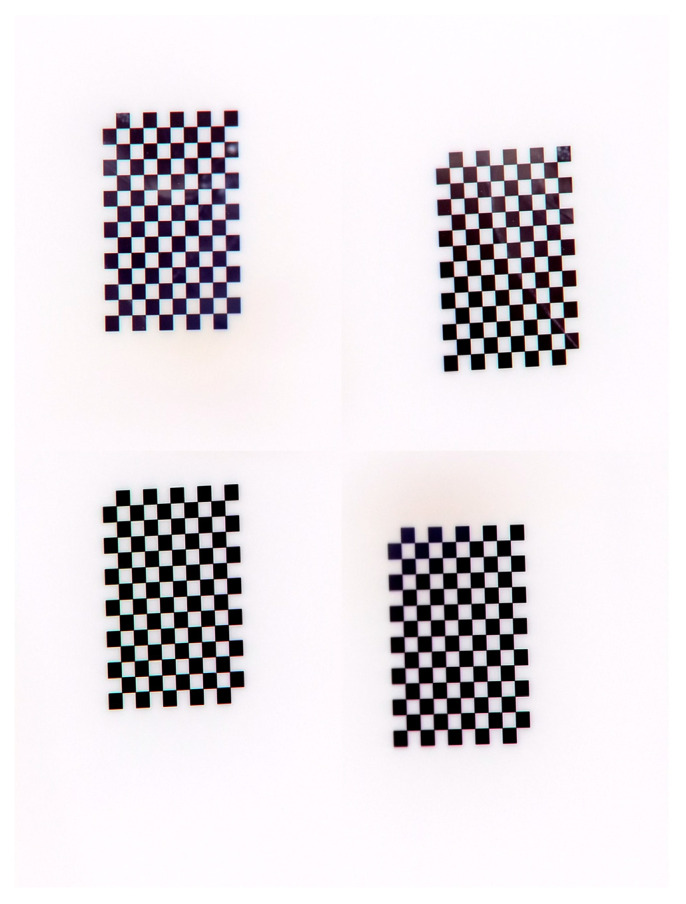
Real images of the calibration board.

The first stage involves the rigorous calibration of the physical telecentric camera array. For statistical validity, a total of 1200 calibration target images were collected. To enhance image contrast and ensure stable feature extraction, a black-white inversion process was applied. A representative captured image of the calibration board is shown in Figure 11.

**Figure 12:**
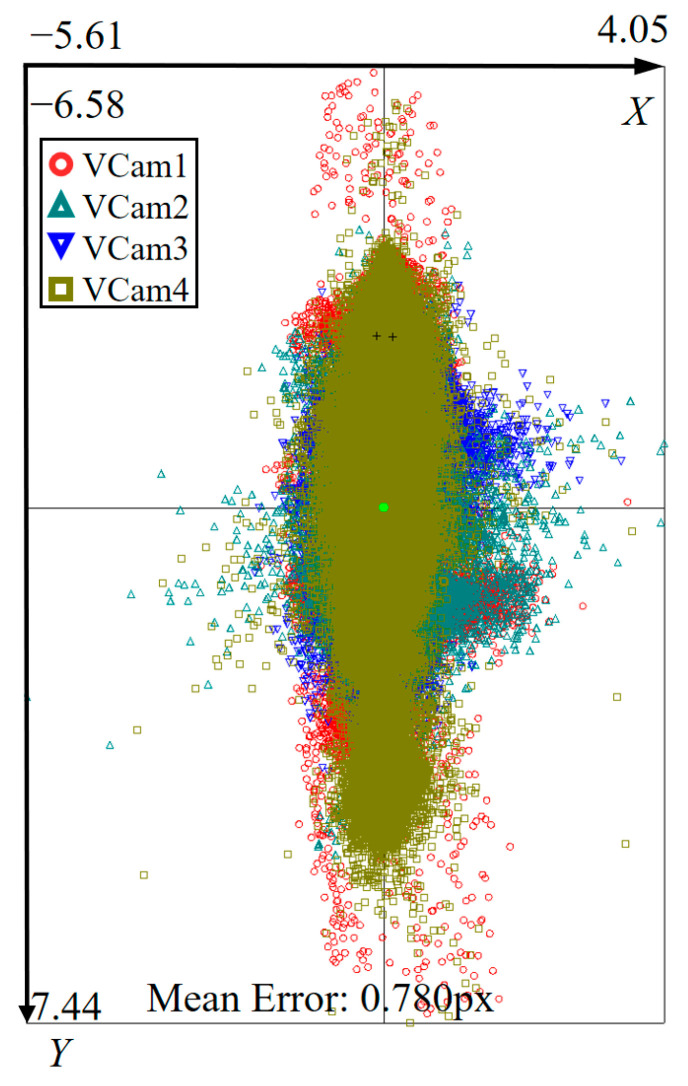
Reprojection error distribution of real telecentric calibration.

After performing the telecentric calibration, the reprojection error distribution was analyzed to assess the internal fitting quality of the mathematical model. As illustrated in Figure 12, the average reprojection error reached 0.78 pixels, which corresponds to an equivalent physical error of approximately 3.432 μm. Given that the virtual camera array achieves an expanded effective field of view of approximately 33.4 mm × 4.27 mm, this micron-scale precision relative to the wide measurement range confirms the high fidelity of the catadioptric telecentric model in characterizing the actual optical path.

### 4.2. Metric Accuracy Verification via Independent Ruler Test

To rigorously evaluate the system’s metric performance using an independent standard, a ruler length measurement test was conducted distinct from the calibration process. This approach avoids data coupling between the calibration and validation datasets, ensuring an objective assessment of the system’s accuracy.

**Figure 13:**
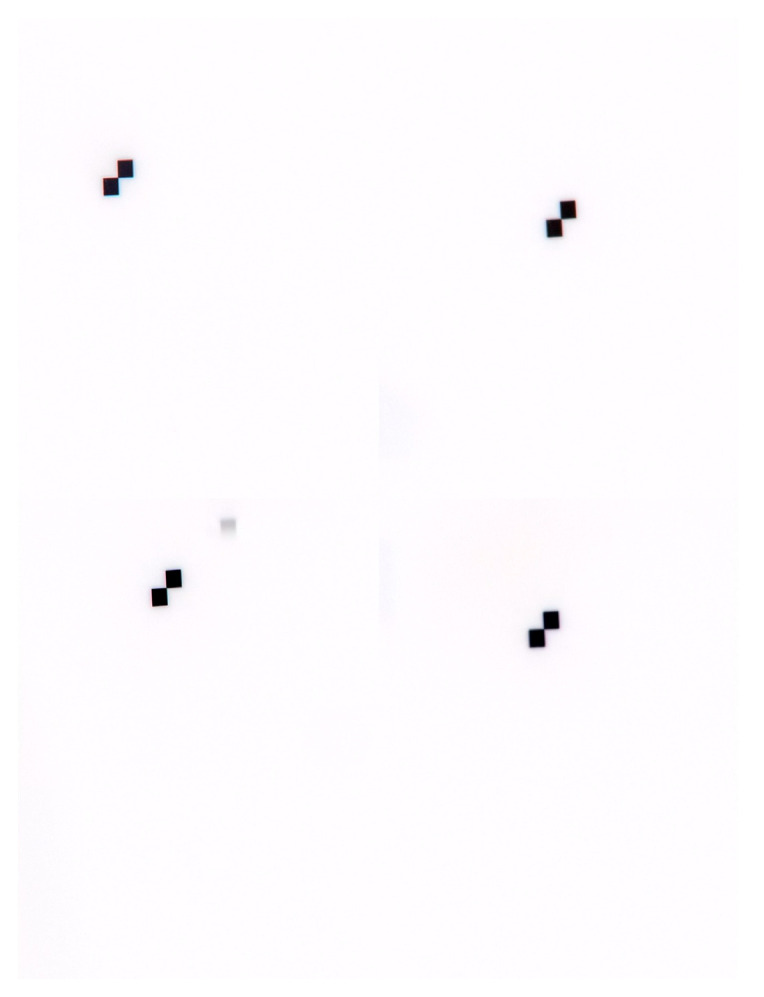
Real images of the high-precision ruler.

**Figure 14:**
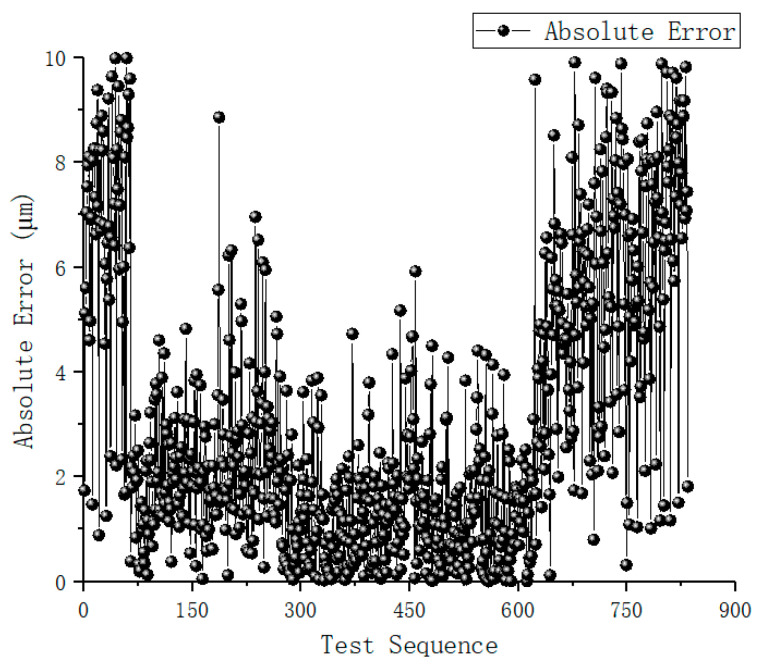
Length measurement error of the real ruler.

A total of 850 ruler images were captured across various poses within the field of view (similar to the capture quality shown in Figure 11). The length measurement error distribution is presented in Figure 13. The average measurement error was found to be 0.612 μm, with a standard deviation of 1.74 μm and a maximum absolute error of 10.02 μm. As illustrated in Figure 14, the error distribution exhibits a characteristic pattern where the precision is highest in the central region and decreases towards the boundaries of the measurement volume.

This ‘U-shaped’ distribution is primarily attributed to the geometric constraints during the sampling process. Since the physical dimensions of the ruler are smaller than those of the calibration board, the latter may not be fully captured at extreme poses (large angles or displacements), leading to less constrained pose estimation in these boundary regions. Furthermore, at the edges of the measurement range, certain optical channels in the four-camera array may approach or slightly exceed the depth-of-field limit. The segmented 1/4 field-of-view of the physical lens acts as an irregular aperture, which tends to intensify non-linear optical aberrations when defocusing occurs. These factors collectively contribute to the larger deviations observed at the ends of the test sequence. Future iterations of the system will focus on optimizing the overlapping depth of field and implementing non-linear error correction models to further enhance global measurement consistency.

### 4.3. Application in Aircraft Pipeline Installation Strain Measurement

**Figure 15:**
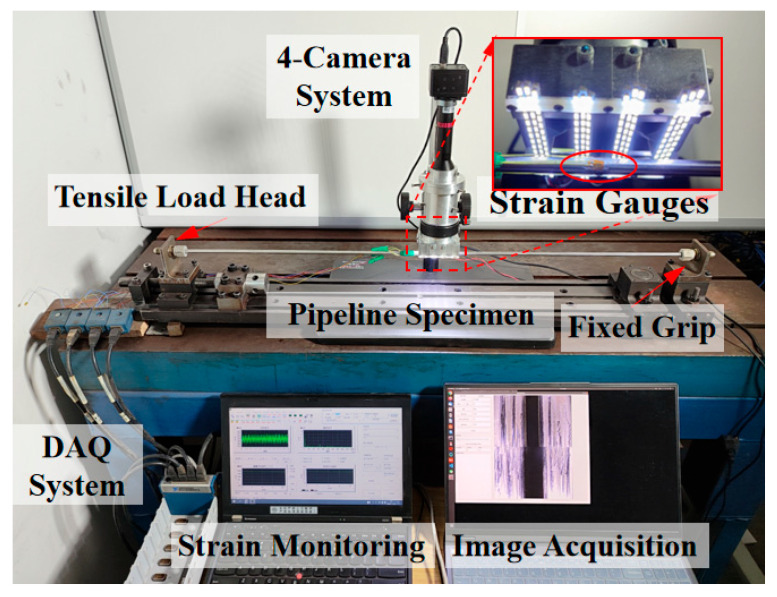
Experimental setup for pipeline strain measurement.

Finally, the validated system was deployed to measure the installation strain of aircraft hydraulic pipelines in a simulated assembly scenario. An experimental test bench was constructed, consisting of a pipeline fixture system for applying axial loads, a strain measurement system using silver-edge strain gauges as a reference, and the telecentric camera array for vision-based calculation, as illustrated in Figure 15.

Regarding the experimental design, a straight-pipe loading configuration was deliberately selected rather than a complex simulated assembly of irregular pipelines. This choice was driven by two primary considerations: First, the utilized multi-axis loading bench allows for the precise quantification and decoupling of loads in specific directions, which is essential for establishing a clear ground-truth for accuracy verification. Second, since the current telecentric system is primarily optimized for axial strain sensitivity, using a straight specimen effectively minimizes the interference from complex, non-linear strain distributions (such as coupled bending or torsion) that often occur in irregular pipe geometries. This approach ensures a rigorous control of variables during this fundamental validation phase.

To extract the strain data from the captured images, the 2D displacement fields of each sub-view were processed using the Ncorr platform in MATLAB 2021. Specifically, a subset size of approximately 500 × 500 pixels (covering roughly 1/4 of the sub-image area) was employed to track feature points across the binocular sub-view sequences. By reconstructing the 3D coordinates from the stereo pairs and monitoring the relative changes in specimen length, the axial strain was derived.

To ensure the stability of the optical system and minimize measurement errors induced by thermal deformation, a preheating protocol was strictly followed. Both the industrial camera and the LED light source were powered on for 10 min prior to the commencement of any tasks. This procedure allowed the system components to reach thermal equilibrium, thereby mitigating potential geometric drifts during the data acquisition process.

**Figure 16:**
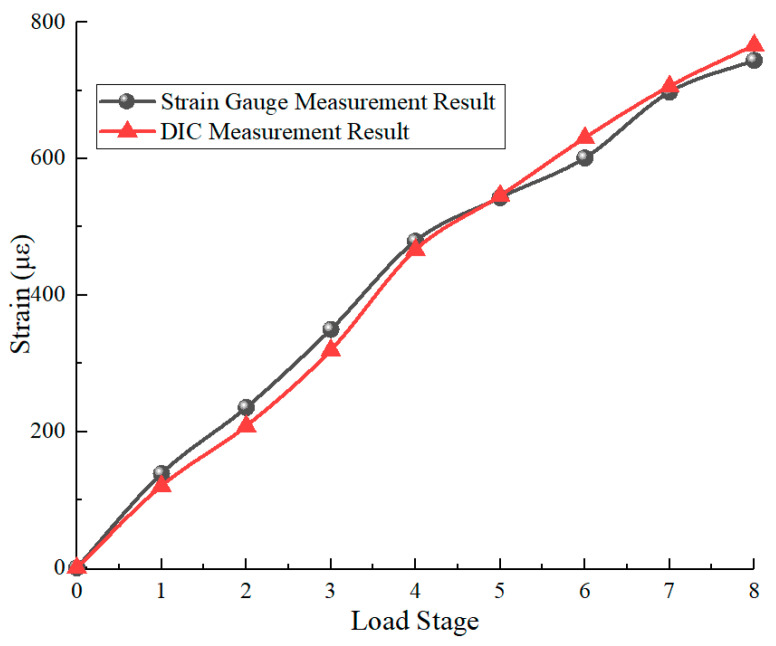
Comparison of Measurement Results Between Telecentric Camera Array and Strain Gauges.

During the experiment, eight loading cycles were applied with the strain ranging from 0 to 750 με. Figure 16 presents the comparison between the strain gauge data and the results from the telecentric camera array. The high degree of consistency between the two datasets verifies the feasibility and reliability of the proposed method for in situ strain monitoring in complex assembly environments.

Regarding the minor discrepancies observed in Figure 16, several qualitative factors contribute to these micro-strain level deviations. First, there is a spatial representation mismatch: the camera array measures the average strain over a 33.4 × 4.27 mm^2^ surface area, whereas the reference value is the arithmetic mean of four discrete strain gauges adhered around the pipe circumference. Local material non-uniformity or processing variances in the pipeline can lead to differences between these localized and area-averaged values. Second, the structural stiffness of the test bench may introduce interference; under high tensile loads, the vertical support plates may undergo slight elastic deformation, potentially causing a minor loss of parallelism that introduces subtle bending effects. Since the camera and gauges occupy different spatial positions, such coupled bending would result in the observed offsets.

## 5. Conclusions

This study develops a compact telecentric camera array for in situ strain measurement of aircraft hydraulic pipelines, with key conclusions as follows.

First, the system’s optical design ensures high axial sensitivity and portability, making it well-suited for the constrained space of aircraft assembly sites. This design addresses the limitations of traditional devices in measuring small components.

Second, both numerical and physical experiments confirm its high precision: the real system achieves an average reprojection error of 0.78 pixels and a ruler measurement error of 0.612 μm. In pipeline strain tests, its results highly align with strain gauges, meeting aircraft hydraulic pipeline assessment requirements.

Third, the non-destructive, non-contact method avoids surface damage or assembly interference from contact techniques (e.g., strain gauges) and overcomes non-contact methods’ low small-component precision, proving practical for on-site monitoring.

Fourth, compared with other optical metrology techniques such as fringe projection, which often require bulky projector-camera setups and are sensitive to ambient lighting, the proposed telecentric array offers a more compact, single-lens solution. Its primary advantage lies in the ability to perform high-precision 3D displacement measurements without requiring the camera to be fixed at a specific global reference position, providing greater flexibility for in situ inspections.

Fifth, while the current prototype is optimized for straight pipeline sections and operates under static or quasi-static conditions, it demonstrates high scalability. The modular catadioptric design can be adapted to various pipe diameters by adjusting the mirror configurations. To maintain measurement reliability under external disturbances such as vibration, future iterations will implement high-frame-rate sensors and accelerated processing algorithms to transition from static checks to real-time dynamic monitoring.

In summary, the telecentric array provides a reliable, high-precision solution for aircraft hydraulic pipeline installation strain measurement and holds potential for other small-scale engineering components. Finally, the limitations of the current optical design were observed. The selected 60° stereo angle resulted in a relatively shallow overlapping depth of field. Although reducing the aperture size compensated for this depth, it inevitably led to a slight degradation in image sharpness. Future work will focus on designing continuous field-of-view measurement systems to accommodate curved or irregular pipeline geometries, as well as incorporating tilt-shift mechanisms to enhance imaging quality.

## Data Availability

The data presented in this study are available on request from the corresponding author.
